# Longitudinal Analysis of Cochlear Implant Stimulation Levels in the First Year: Optimizing Adult Fitting

**DOI:** 10.1097/AUD.0000000000001875

**Published:** 2026-08-19

**Authors:** Maryam Hussain, Hylke F. E. van der Toom, Mats Voogelaar, Bernd Kremer, Jantien L. Vroegop

**Affiliations:** 1 Department of Otorhinolaryngology and Head and Neck Surgery, Erasmus University Medical Center, Rotterdam, the Netherlands; 2 Department of Ophthalmology, Erasmus University Medical Center, Rotterdam, the Netherlands; 3 The Rotterdam Eye Hospital, Rotterdam, the Netherlands; 4 Department of Epidemiology, Erasmus University Medical Center, Rotterdam, the Netherlands.

**Keywords:** Adults, Cochlear implant, Linear mixed model, Postlingual, Rehabilitation, Stimulation levels

## Abstract

**Objectives:**

The main objective of this study was to investigate the evolution in stimulation levels (C-/M- and T-levels) in adult cochlear implant users from both Advanced Bionics and Cochlear, and to assess whether this trajectory differs between the two brands. The secondary objective was to assess individual patterns of stimulation levels during the first year post-implantation.

**Design:**

This retrospective study was conducted at the Erasmus University Medical Center in Rotterdam, the Netherlands. A total of 183 postlingually deaf adults with a unilateral cochlear implant from either Advanced Bionics or Cochlear were included. Stimulation levels (C-, M-, and T-levels) were collected from these adults for six fitting sessions during the first year postimplantation. Linear mixed-effects models were used to analyze longitudinal changes.

**Results:**

Our findings were comparable for both Advanced Bionics and Cochlear implant users. Mean M/C and T levels changed over time, showing a similar characteristic pattern for each patient with a rapid increase during the initial period, followed by gradual stabilization. Post hoc comparisons revealed significant differences between appointments up to the fourth fitting session for M/C levels and up to the third fitting session for T-levels. There were no significant differences after these fitting sessions. These findings were consistent across the different electrode segments (apical, medial, upper basal, lower basal), although earlier stabilization occurred in the lower basal segment for M/C-levels and in both basal segments for T-levels.

**Conclusions:**

The present study gave valuable insights into the temporal evolution of M/C- and T-levels during the first year postimplantation for both Advanced Bionics and Cochlear implant users. The mean M/C- and T-levels stabilized after approximately 3 months and 5 weeks, respectively, showing equivalent trajectories between the two brands. A consistent pattern was observed across patients, with stimulation levels rising significantly in the first few months and gradually stabilizing over time.

## INTRODUCTION

Hearing loss is among the most prevalent and undertreated disabilities worldwide (Shen et al. 2025). For many individuals with severe to profound hearing loss, a cochlear implant (CI) can be a life-changing option (Naples & Ruckenstein 2020). A CI converts sound into electrical signals and transmits these signals to the auditory nerve via an electrode array implanted in the cochlea. Cochlear implants have been shown to successfully restore auditory perception, enabling many recipients to understand speech, while also improving overall quality of life (Wilson et al. 1991; Wilson & Dorman 2008). As the benefits of CIs become increasingly recognized, the number of candidates for cochlear implantation is expected to rise significantly in the coming years. This growth is driven by both demographic changes and the expansion of eligibility criteria (Colby & Ortman 2015; Nijmeijer et al. 2023). Consequently, this growing demand places increasing pressure on cochlear implant centers.

The CI care pathway consists of three distinct phases: a selection phase, a rehabilitation phase, and follow-up care. As the rehabilitation phase typically requires numerous fitting sessions and therefore considerable clinical resources, this paper focuses specifically on this stage of care. During the rehabilitation phase, the speech processor is fitted and gradually adjusted to optimize auditory outcomes, while recipients undergo a process of adapting to the device and practicing hearing in real-world environments (Vaerenberg et al. 2014). In the fitting process, the primary goal is to define two stimulation levels: the upper electrical current level (C- or M-level, depending on the manufacturer) that can be comfortably tolerated and the lower electrical current level (T-level) at which sound becomes audible (Shapiro & Bradham 2012; Wolfe & Schafer 2025). Together, these two levels define the patient’s electrical dynamic range within which all stimulation levels during device use are set.

The cochlear implant device is typically activated approximately 2 to 4 weeks post-surgery (Shapiro & Bradham 2012; Vaerenberg et al. 2014). The initial fitting of a cochlear implant takes place during the first activation session, where baseline stimulation levels are established. In the following weeks, multiple fitting sessions are required to optimize stimulation levels, as studies have reported significant changes in these levels over time in both children and adults (Hughes et al. 2001; Henkin et al. 2003; Vargas et al. 2012; Mosca et al. 2014). Most of these changes occur in the first year after activation due to various factors such as intra-cochlear fibrosis and better patient adaptation to the new type of stimulation (Kawano et al. 1998; Hughes et al. 2001). Studies have consistently demonstrated that stimulation levels evolve quickly during the initial weeks after activation, followed by a slower progression and eventual stabilization. Specifically for adults, studies have found that T-levels can stabilize within 2 to 3 months after activation (Hughes et al. 2001; Mosca et al. 2014). In contrast to T-levels, more variation is found for the upper current levels. These studies have also shown varying times for upper current levels to stabilize after cochlear implant activation. While Mosca et al. (2014) indicated that stabilization occurs approximately three months post-activation, some suggest the process may extend to six months or 1 year (Hughes et al. 2001; Vargas et al. 2012). These studies include relatively small groups of postlingually deaf adults, ranging from 19 to 33 adults, which may explain the differences in results. Two more recent studies by Gajadeera et al. (2017a) and Dornhoffer et al. (2025) included substantially larger samples of 508 and 428 postlingually deaf adults with Cochlear CIs, respectively, thereby offering greater statistical power and more robust insights. Both studies demonstrated that C-levels stabilized by six months post-activation. Gajadeera et al. observed T-level stabilization after 3 months, which is in line with previous research reporting T-level stabilization within 2 to 3 months, whereas Dornhoffer et al. observed stabilization after 12 months, which contradicts earlier findings. Moreover, the amount and timing of fitting sessions during the rehabilitation phase varied across both smaller and larger studies.

Despite these valuable insights, no standardized fitting protocol has emerged regarding the amount and timing of fitting sessions, leading to large variation in the fitting practices between centers (Vaerenberg et al. 2014; Browning et al. 2020). Moreover, nearly all previous studies investigating the evolution of stimulation levels during the first year postimplantation have included only Cochlear CIs, thereby limiting the generalizability of their findings to other CI brands. No comparable analysis has been performed on the evolution of stimulation levels for Advanced Bionics (AB) implants. Furthermore, many of these studies have been conducted with relatively small sample sizes, and previous studies did not apply appropriate statistical methods capable of accounting for intra-individual variability in repeated measurements, such as linear mixed models (LMMs). This leaves a gap in our understanding of how fitting strategies work across different devices and larger, more diverse cohorts (Dornhoffer et al. 2023).

In our hospital, the rehabilitation phase includes six fitting sessions during the first year after implantation. Fitting sessions are essential but can be time-consuming, contributing to the increasing workload faced by CI centers. To optimize the current rehabilitation process, the present study aims to evaluate the current fitting strategy within our center to determine whether a more efficient approach can be developed without compromising the quality of care. Compared with previous studies, the contribution of our study is that (1) it analyzes data from two different CI brands, thereby broadening generalizability beyond previous single-brand studies; (2) it performs a more robust statistical analysis that accounts for intra-individual variability; (3) it involves a relatively large cohort of CI recipients.The primary objective of this study was to investigate the evolution of C-/M- and T-levels of adult CI users during the first year postimplantation and to assess whether this evolution differs between AB and Cochlear CI users. The secondary objective was to evaluate the pattern of patient-specific stimulation levels over time within the first year postimplantation. By identifying patterns in the stabilization process of the stimulation levels, we aim to determine whether it is possible to enhance efficiency without compromising care quality.

## MATERIALS AND METHODS

### Participants

A retrospective case review was conducted at the Erasmus University Medical Center in Rotterdam, the Netherlands. All adult patients (≥18 years) with bilateral severe-to-profound postlingual hearing loss who received a unilateral CI from either AB or Cochlear between January 1, 2019 and February 29, 2024, at the Erasmus University Medical Center were considered for inclusion in this study. Postlingual hearing loss was defined as hearing loss with an onset after the age of 3 years.

The following exclusion criteria were applied: (1) CI failure, (2) incomplete insertion, (3) non-user (<3 hours of device usage), (4) reimplantation, (5) bilateral CI implantation, (6) incomplete rehabilitation within the first year due to relocation, mortality, or other factors, (7) restart of rehabilitation due to limited CI functioning, (8) atypical responses on certain electrodes, (9) involved in other ongoing research, (10) variation in pulse width settings during fitting sessions within the first year (this variation influences the range of M/C- and T-levels, making reliable comparisons across fitting sessions impossible).

During the study period, 254 adult patients received a CI, either from AB or Cochlear. Twenty-five patients underwent reimplantation, most of which were performed following a Voluntary Field Action by one of the manufacturers due to device failure. Furthermore, six patients were bilaterally implanted, two patients were non-users, and three patients did not complete their rehabilitation program because they passed away within their first year after implantation. One patient was excluded because the rehabilitation phase was started for the second time, and one patient had atypical responses on certain electrodes. Two patients were excluded because they were part of an ongoing scientific research project. Thirty-one patients who had a Cochlear CI were also excluded because they were fitted with varying pulse widths across the fitting sessions. A total of 183 eligible patients were ultimately included in this study, comprising 94 patients with AB CIs and 89 patients with Cochlear CIs. An overview of the patient selection process is presented in Fig.

**Fig. 1.**
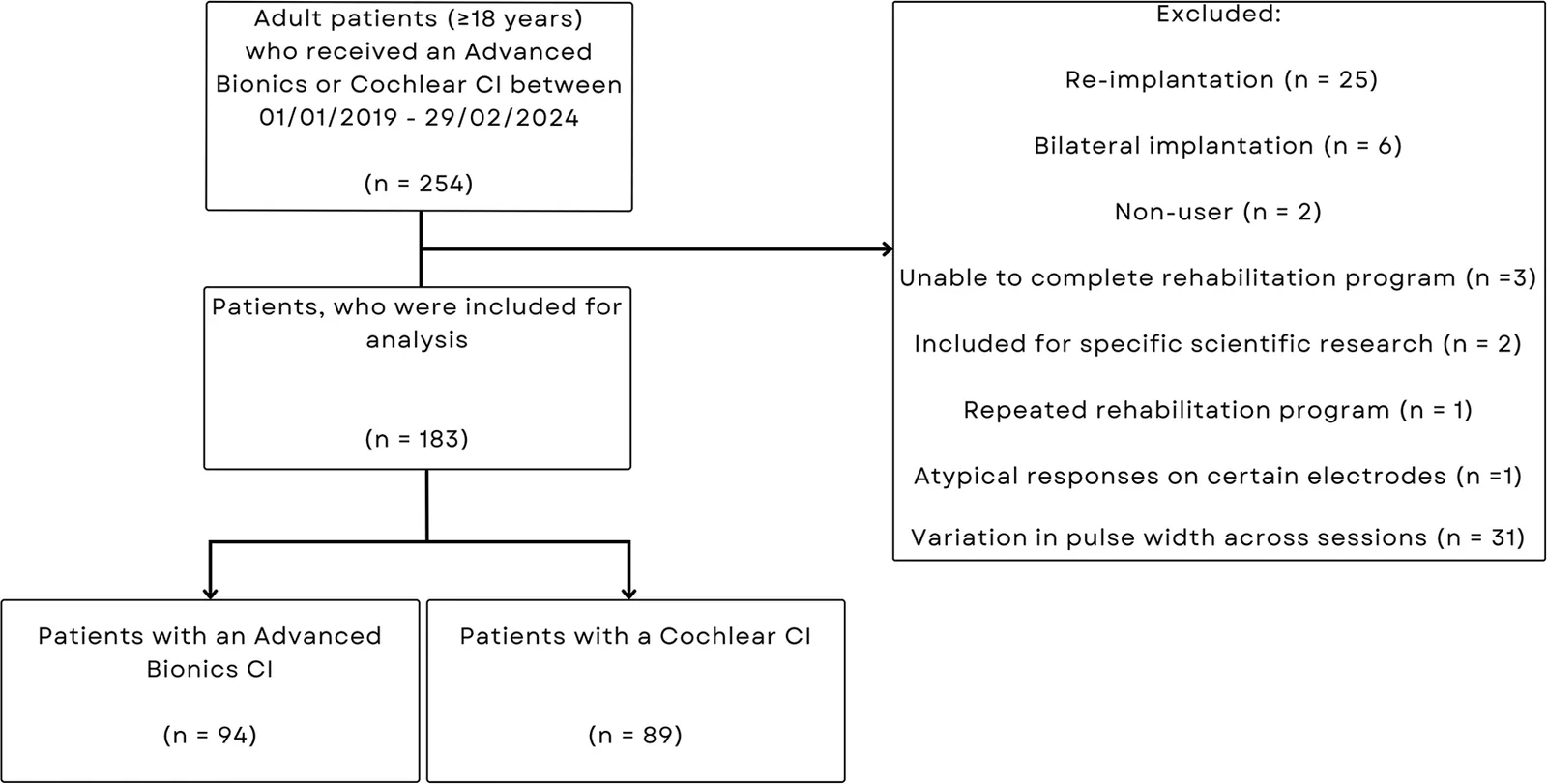
Flowchart of patient selection, based on the inclusion and exclusion criteria.

### Procedures

#### Fitting Process

Stimulation levels (C-, M-, and T-levels) for each electrode were retrospectively collected per appointment from cochlear implant fitting software: Soundwave 3 or Target CI for AB CIs and Custom Sound Pro for Cochlear CIs. Both brands refer to the lower level of the dynamic range as the threshold level (T-level). The T-level is generally set near the point at which an auditory sensation begins to occur. Depending on the fitting strategy, this may be defined as the lowest perceivable level or just below the level at which a sound is detected. For the upper level, AB and Cochlear use different terminology. AB uses the term M-level and defines the upper limit as the most comfortable stimulation that can be perceived, while Cochlear uses the term C-level and defines the upper limit as the maximum comfortable level that can be perceived.

In our clinic, the CI team consists of five audiologists, who follow a detailed and specified protocol regarding CI care and specifically the fitting process. Both AB and Cochlear have different fitting processes and fitting software. For AB CIs, Soundwave 3 or Target CI is used as fitting software, and Custom Sound Pro is used for Cochlear CIs. Before fitting, impedances are measured and checked for CIs from both brands. For AB CIs, at activation, the M-levels are first established by measuring the M-levels using a speech burst. After this, electrodes are measured with a tone burst. T-levels are initially set at 10% of the M-levels by default. Subsequently, the overall M‑levels are reduced, and the program is activated. The M-levels are further adjusted while speaking to the patient, aiming for speech to be comfortably loud. Progressive maps are made with a step size of 10 current units. T-levels are adjusted during activation or following fitting sessions using an ascending presentation followed by a standard bracketing procedure.

For Cochlear CIs, at activation, T-levels are first acquired through an ascending presentation, followed by a standard bracketing procedure. The threshold is set at the lowest T-level at which a response is observed. After initial T-levels are determined, the T-level profile is temporarily reduced by 10 current levels to prevent the perception of unintended sounds in silence during initial activation. The program is then activated, and C-levels are gradually increased until the patient indicates that speech reached the maximum level that is still comfortably loud. If C-levels are just above the T-levels and speech remains uncomfortably loud, both C-levels and T-levels are lowered. In the end, an attempt is made to increase T-levels to their originally measured levels. Progressive maps are made with a step size of five current level.

During follow‑up fittings, for both brands M‑, C- and T-levels are further adjusted based on audiometric assessments and patient feedback. The audiometric assessments include free field pure tone audiometry and free field speech audiometry and are conducted at the second, third, and fourth fitting sessions and at 1-year. Free field speech audiometry is used to evaluate the patient’s auditory performance and to monitor progress throughout the rehabilitation. The measured free field pure tone audiometry is used to evaluate and identify which frequency regions require additional stimulation.

Patient feedback is a subjective measure used to fine-tune the T-, M-, and C-levels. It provides information on perceived loudness, sound quality, and listening comfort, and is used to prevent loudness discomfort and optimize the fitting.

#### Fitting Scheme of Rehabilitation Phase

The current standard rehabilitation phase at the Erasmus MC follows a protocol that schedules six fitting sessions during the first year within specific time periods. These time periods are: T_1_ (switch on), T_2_ (1 to 2 weeks after switch on), T_3_ (3 to 6 weeks after switch on), T_4_ (9 to 14 weeks after switch on), T_5_ (approximately 6 months after switch on), and T_6_ (approximately 12 months after switch on). In practice, appointments often deviate from these scheduled time periods, so acceptable time ranges were defined: T2 (1 to 3 weeks), T3 (>3 weeks to 1.5 months), T4 (>1.5 to 4 months), T5 (4 to 9 months), and T6 (>9 to 15 months). Electrodes were assigned to lower basal (E14 to E16 for AB, E1 to E4 for Cochlear), upper basal (E11 to E13 for AB, E5 to E8 for Cochlear), medial (E6 to E10 for AB, E9 to E15 for Cochlear), and apical (E1 to E5 for AB, E16 to E22 for Cochlear) segments of the intracochlear coil.

### Data Analysis

The evolution of C-, M-, and T-levels over time was visualized per patient. Mean C-, M-, and T-levels were calculated across all electrodes per corresponding time point and per segment (lower/upper basal, medial, and apical). These mean values were used to conduct linear mixed-effects models (Laird & Ware 1982). These models allow accounting for repeated measurements within participants. In these models, fixed effects represent the effect of the predictor (time) on the outcome (stimulation level), while random effects capture variability between participants. The linear mixed-effects models were specified as: *y* = *Xβ* + *Zu* + *e*. The measured stimulation level *y* is defined as the sum of the overall time-dependent trend *β*, the individual patient’s unique trajectory *Zu*, and a small residual error term *e.*

First, the overall effect of time on the stimulation levels was examined by including the timepoint order as a numeric fixed effect, yielding a *β* representing the average change per appointment. To account for individual differences, a random intercept and slope for the timepoint for each CI user was included, allowing individual differences in baseline levels and rates of change.

Second, time was modeled as a categorical fixed effect (6 timepoints) with the same random-effects structure to evaluate differences between the individual timepoints using post hoc pairwise comparisons, which addressed our primary research objective. The fixed-effect *β* coefficients from these mixed models were not used for interpretation, as they represent differences relative to a single reference timepoint only.

Instead, post hoc pairwise comparisons between timepoints were obtained using Tukey’s honestly significant difference test (Tukey 1949). This test compares the differences between all pairs of estimated marginal means and adjusts for multiple testing to control the type I error rate (false positives) (Searle et al. 1980). Model assumptions were evaluated by plotting the conditional residuals of the mixed-effects models against the fitted values and using Q–Q plots. Residuals showed no evidence of heteroscedasticity, and Q–Q plots indicated normality, indicating that model assumptions were met. A significance threshold of *p* < 0.05 was applied. Estimated marginal means with 95% confidence intervals were also plotted to visualize the evolution of stimulation levels across timepoints. Analyses were performed in RStudio, using R version 4.5.0.

## RESULTS

### Demographic Factors

A total of 183 eligible patients were included in this study for analysis, comprising 94 patients with AB CIs and 89 patients with Cochlear CIs. Mean age at the time of implantation was 66.3 years (SD: 14.7 years; range: 24 to 90 years) for patients with AB CIs and 63.2 years (SD: 17.2 years; range 18 to 91 years) for patients with Cochlear CIs. Multiple types of implants were used: HiRes Ultra 3D MS (n = 94) in the AB group and Nucleus CI612 (n = 51), Nucleus CI632 (n = 23), Nucleus CI24RE Freedom (n = 10), and Nucleus CI532 (n = 5) in the Cochlear group. The number of CI patients attending each scheduled fitting session during the first year after implantation is presented in Fig. 2, separately for AB and Cochlear. The fitting sessions of the patients within and outside the predefined time ranges, and the missing fitting sessions, for both Cochlear and AB can be viewed in the supplemental files (Tables S1a and S1b in Supplemental Digital Content, https://links.lww.com/EANDH/B995).

**Fig. 2.**
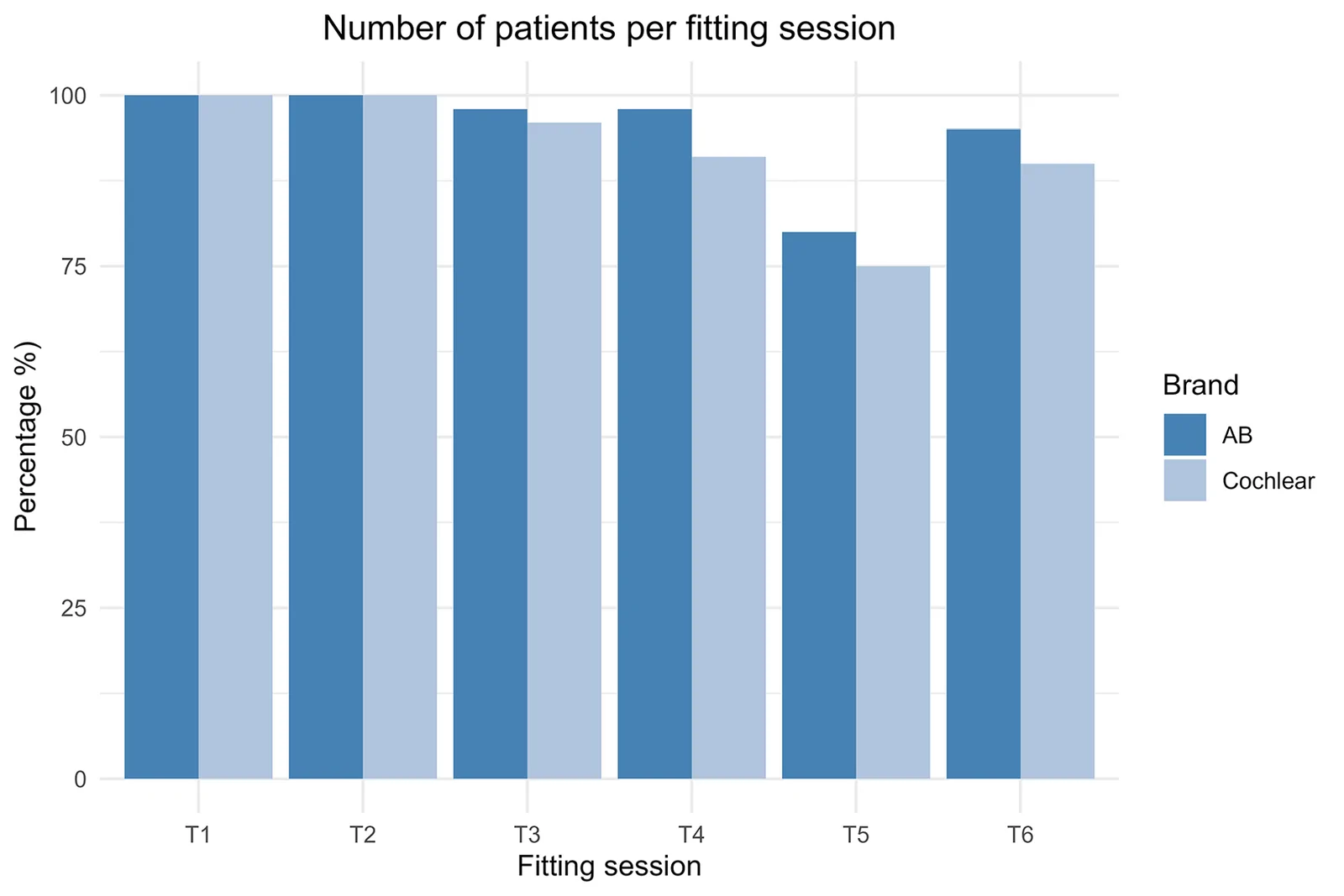
Patient participation across the six standard fitting sessions during the first year postimplantation.

### Evolution of the Stimulation Levels Across All Electrodes Per Appointment

Tables 1 and 2 show the mean stimulation levels across all electrodes for all patients at each timepoint with corresponding standard errors. These tables show that stimulation levels gradually increased over the appointments for both AB CI users and Cochlear CI users, with the most pronounced increase occurring between T1 and T2. From T4 onward, the C- and M-levels started to stabilize across sessions, while T-levels showed relative stabilization from T3 onward. The standard error of the mean was larger for the stimulation levels of patients with an AB CI compared with those of patients with a Cochlear CI.

**TABLE:** 1. Mean T- and M-levels of all patients with an Advanced Bionics CI

	Mean M-Level (Overall)	SEM	Mean T-Level (Overall)	SEM
T1	102.5	3.4	23.4	2.3
T2	137.4	3.5	37.9	1.5
T3	160.1	3.4	42.5	1.8
T4	170.4	3.6	44.9	2.2
T5	170.8	4.2	46.0	2.8
T6	174.7	3.8	46.7	2.3

CI = cochlear implant.

**TABLE 2.** Mean T- and C-levels of all patients with a Cochlear CI

	Mean C-Level (Overall)	SEM	Mean T-Level (Overall)	SEM
T1	131.3	1.9	105.3	1.9
T2	150.6	1.7	114.6	1.8
T3	160.9	1.7	119.4	1.9
T4	165.0	1.8	120.6	1.8
T5	166.7	2.2	123.1	2.2
T6	166.1	2.0	121.1	2.0

CI = cochlear implant.

For patients with AB CIs, the linear mixed-effects model showed significant changes in stimulation levels over time for both M-levels (*β* = 13.8, SE = 0.7, *p* < 0.001) and T-levels (*β* = 4.0, SE = 0.6, *p* < 0.001), indicating variation across the six appointments. For patients with Cochlear CIs, the linear mixed-effects model also showed significant changes in stimulation levels over time for both C-levels (*β* = 6.6, SE = 0.4, *p* < 0.001) and T-levels (*β* = 3.0, SE = 0.3, *p* < 0.001). Post hoc tests were conducted to explore specific pairwise differences between the timepoints. The results of this analysis are shown in Table S2a for the AB group and Table S2b in Supplemental Digital Content, https://links.lww.com/EANDH/B996, for the Cochlear group. In both groups, there were no significant differences in C/M-levels observed from T4 onward. For the T-levels, no significant differences were observed from T3 onward.

To provide an overview of the time-dependent changes in average stimulation levels for each appointment, estimated marginal means are visualized in Fig. 3A, B for the AB group and in Fig. 3C, D for the Cochlear group. This figure also indicates between which consecutive appointments a significant difference exists.

**Fig. 3.**
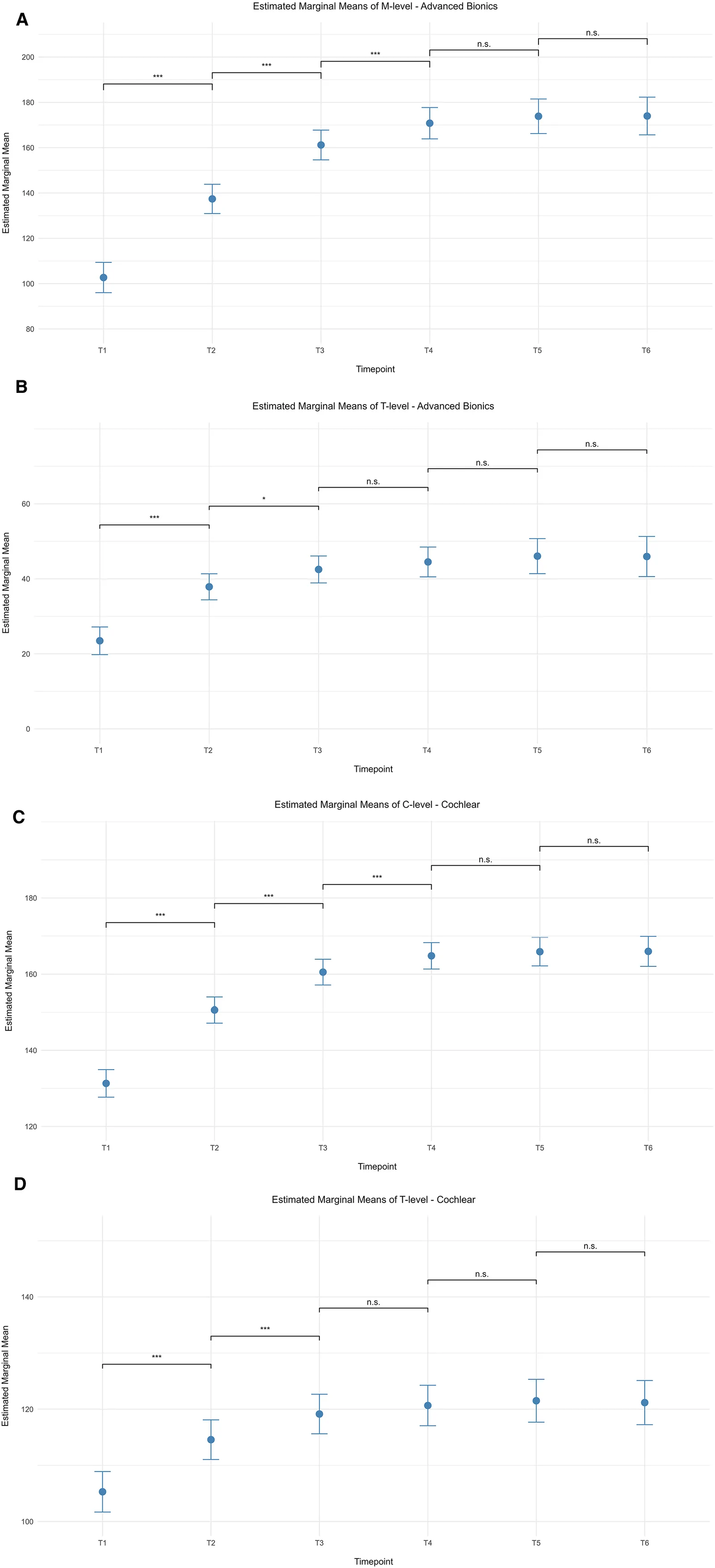
Estimated marginal means with confidence interval across six timepoints for M- and T-levels in the Advanced Bionics group and C- and T-levels in the Cochlear group. ***p < 0.001; n.s. indicates not significant.

### Evolution of Stimulation Levels Per Segment Across Appointments

Post hoc tests were also conducted to evaluate specific pairwise differences between timepoints within each cochlear segment (apical, medial, upper basal, and lower basal), separately for each stimulation level for both AB and Cochlear. A detailed overview of these post hoc tests is provided in Tables S3a and S3b in Supplemental Digital Content, https://links.lww.com/EANDH/B997, for the AB group and Tables S4a and S4b in Supplemental Digital Content, https://links.lww.com/EANDH/B998, for the Cochlear group.

For both the AB group and the Cochlear group, the M/C-levels of the apical, medial, and upper basal segment showed no significant differences from T4 onward. The M/C-levels of the lower basal segment showed no significant differences from T3 onward. For both the AB group and the Cochlear group, the T-levels of the apical and medial segment showed no significant differences from T3 onward. For the upper and lower basal segment, no significant differences were observed from T2.

### Longitudinal Changes of Patient-Specific Stimulation Levels

Fig. 4A, B show the evolution of the M- and T-levels per patient over time in days for the AB group, and Fig. 4C, D show the evolution of the C and T-levels per patient for the Cochlear group. Across individual trajectories, C/M- and T-levels showed a comparable pattern of change in stimulation levels over time. These figures show a clear increase in stimulation levels in the beginning, after which the stimulation levels appear to start stabilizing between approximately day 50 and day 100.

**Fig. 4.**
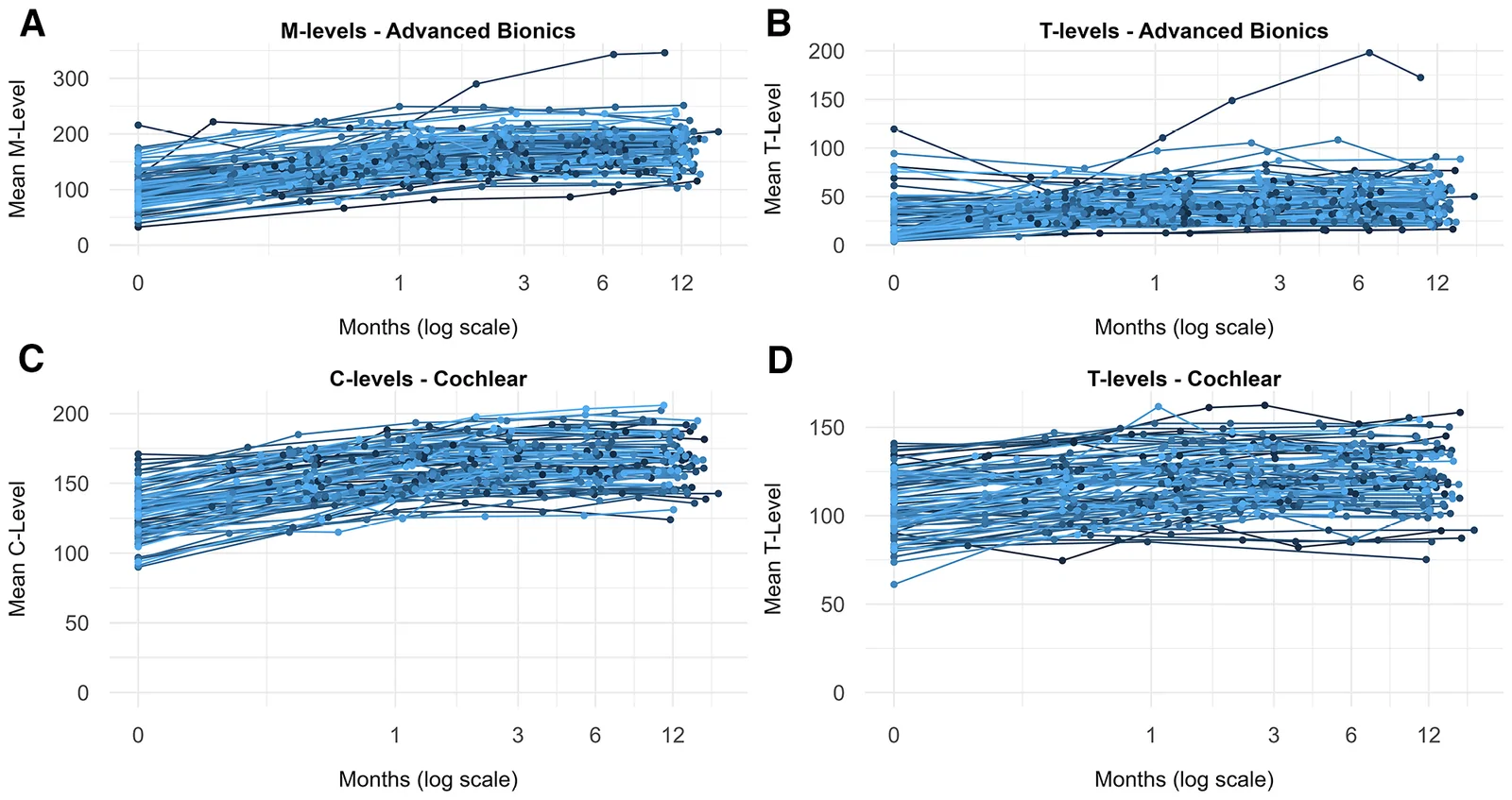
Development of individual stimulation levels over time for both Advanced Bionics and Cochlear implant users.

## DISCUSSION

### Evolution of Stimulation Levels

The present study evaluated the evolution of stimulation levels of 183 postlingually deaf adult CI users during the first year after activation. Our findings showed that stimulation levels gradually increase over time but eventually stabilize, with the largest increases occurring during the first month postimplantation. Mean C/M-levels were found to stabilize from approximately 3 months post-activation, whereas the mean T-levels began to stabilize earlier, approximately 5 weeks after activation. These results were consistent for both AB and Cochlear implant users. This suggests that most patients reach their target stimulation range relatively early in the fitting process. In general, this pattern of the evolution of stimulation levels remained consistent across segments. However, in the lower basal segment, C/M-levels stabilized earlier, from T3 instead of T4. Similarly, T-levels in both lower and upper basal segments showed earlier stabilization, from T2 rather than T3. Thus, there are some segment-specific differences in the timing of level stabilization. Although AB and Cochlear have different fitting and stimulation methods, especially for measuring the T-levels, similar patterns were observed across devices. This gives meaningful insights that, despite the methodological differences between the two brands, the functional outcome appears to align, and thus the stimulation levels stabilize at similar timepoints.

Our findings are consistent with previous studies (Hughes et al. 2001; Mosca et al. 2014), especially regarding the T-levels, showing that significant changes in stimulation levels occur within the first few fitting sessions. Gajadeera et al. (2017a), the largest cohort to date, observed stabilization of C-levels from 6 months and T-levels from 3 months, while Dornhoffer et al. (2025) observed stabilization after 6 and 12 months, respectively. While their findings indicate stabilization of C-levels from 6 months, our results show that stabilization occurred earlier. Nevertheless, the overall pattern across the subsequent time points is similar. Moreover, the segment-specific findings were also similar, demonstrating more rapid stabilization in the lower and upper basal segments. This observation has important implications for the fitting process. It indicates that channel balancing in the basal electrodes is relatively more reliable during early fitting sessions (Sainz et al. 2003). In contrast, apical and medial electrodes, which stabilize later, may require additional adjustments in subsequent fitting sessions to achieve a consistent loudness profile across all electrodes.

It is interesting that our protocol included six scheduled appointments within the first year postimplantation, similar to the recent study by Dornhoffer et al. (2025). Gajadeera et al. (2017a), by contrast, implemented five appointments. Moreover, both Dornhoffer et al. and Gajadeera et al. included a fitting appointment at 9 months, which is not included in our fitting protocol. Instead, our study had an additional early follow-up at 2 weeks post-activation. These differences in scheduling may explain the earlier stabilization of C- and T-levels observed in our cohort, possibly indicating that earlier fitting sessions are associated with faster stabilization of stimulation levels. It is important to note that both studies were limited to Cochlear CIs, unlike our study, which included both AB and Cochlear implant users.

### Longitudinal Changes of Patient-Specific Stimulation Levels

In addition to the overall analysis, the temporal pattern of C/M- and T-levels was explored more directly by evaluating each patient’s stimulation profile over time. This approach allowed identification of trends that may not be apparent in the average group. This profile, for both C/M and T-levels in Cochlear and AB implant users, reflected a similar trajectory of early increase followed by stabilization, which was consistent across patients. Nonetheless, some variability was observed across patients in the evolution patterns and timing of stabilization. Although these individual differences were accounted for in the statistical analysis, this highlights the importance of examining patient-specific trajectories, which is crucial for accurate interpretation and guiding future personalized care strategies.

### Strengths and Limitations

A key strength of our study is that it is the first to investigate the evolution of the stimulation levels of AB implant users. Prior studies have been restricted to Cochlear CIs, whereas we included two different brands, Cochlear and AB. This enhances the generalizability of our findings. In addition, our study is the first to use LMMs to analyze the evolution of stimulation levels during the first year postimplantation. This statistical approach allows the inclusion of both fixed effects, which estimate the average effect across the study population, and random effects, which account for intra-individual variability (West et al. 2022). This makes it possible to model repeated measurements within the same individuals over time, handle missing data effectively, and thus provide more precise estimates of change. Moreover, this study included one of the largest cohorts of adults with a cochlear implant, exceeded only by the studies conducted by Gajadeera et al. (2017a) and Dornhoffer et al. (2025). Other previous research has been based on substantially smaller sample sizes. Furthermore, our study evaluated the evolution of stimulation levels within a fitting protocol that differed from the two previous large-scale studies.

Despite these strengths, some limitations should be considered. It is important to note that our study only focused on changes in stimulation levels within the first year postimplantation. Therefore, it cannot be concluded that the found stability of the stimulation levels persists after 1 year postimplantation. Other research (Gajadeera et al. 2017b; Zinfollino et al. 2020) has shown that for some patients in the long term, after the first year of implantation, adjustments to the stimulation levels can still improve speech perception, with significant differences observed in C and T-levels. These effects may result from intracochlear changes affecting the interface between electrodes and neural tissues. Moreover, a potential limitation is that the effect of individual audiologists on cochlear implant fitting was not included in the statistical analysis. Patients are treated by multiple audiologists during their rehabilitation trajectory, and differences in CI programming cause inter-audiologist variability. However, only five audiologists were involved, and all fittings were conducted according to a standardized clinical protocol. Therefore, we expect the influence of inter-audiologist variability on the results to be minimal. In addition, clinically relevant outcomes, such as speech perception scores, were not assessed. Previous research has shown that average Consonant-Nucleus-Consonant word scores stabilized after 3 months, with the mean word recognition performance expected to fall between 47.2% and 51.5% (Grisel et al. 2022). The observed stabilization at this timepoint aligns with our findings. This suggests a potential similarity between the stabilization of stimulation levels and speech outcomes. This would be interesting and valuable to investigate in further research. In addition to future research, it is valuable to investigate how to optimize the fitting protocol. Aside from the stabilization of the stimulation levels over time, we observed that approximately 25% of CI users did not have a scheduled appointment at T5, as their rehabilitation progress was considered sufficient and no further adjustments were deemed necessary. This finding suggests that implementing remote tools during the rehabilitation phase could be promising. The need to improve efficiency has been addressed in the past, resulting in the introduction of the Fitting to Outcome eXpert system, which was developed to streamline CI fitting by providing automated level optimization. Use of this system contributed to a more efficient fitting process, requiring fewer fitting sessions. However, manual mapping is still commonly preferred. Nevertheless, the Fitting to Outcome eXpert system remains relevant as a reference model for streamlining the fitting process.

In conclusion, our study demonstrates that M/C-levels reached stability around 3 months, whereas T-levels stabilized earlier at approximately 5 weeks post-activation. These findings were consistent for both AB and Cochlear implant users. Each patient exhibited a similar pattern of stimulation level changes over time, namely a substantial increase during the first three months post-activation and then starting to stabilize. Thus, our findings serve as a foundation for future research to optimize the current fitting strategy and develop a more streamlined care pathway for adults with a cochlear implant within the first year postimplantation. These findings highlight the importance of multiple fitting sessions in the first months after CI implantation. Furthermore, the stimulation levels stabilize after a few months, regardless of the type of CI brand. This stabilization supports considering re-evaluation of the current CI care pathway, allowing for a more streamlined CI care pathway, including the implementation of remote monitoring.

## ACKNOWLEDGMENTS

This research was funded by the Immunis Foundation (Stichting Immunis, Reeuwijk). The funding organization had no role in the design and conduct of the study; in the collection, analysis, and interpretation of the data; or in the decision to submit the article for publication; or in the preparation, review, or approval of the article.

## Supplementary Material
