# Supplementary material for: Longitudinal Analysis of Cochlear Implant Stimulation Levels in the First Year: Optimizing Adult Fitting
Source: Ear Hear. 2026 Aug 19;47(6):1685–91. doi: 10.1097/AUD.0000000000001875 (PMC13712652; doi:10.1097/AUD.0000000000001875)
Supplement: Supplementary file 1 [file aud-47-1685-s001.pdf]

Supplemental data file

Table S1a and S1b show the number of patients and their corresponding appointments within the predefined time range, the number of patients and their corresponding appointments outside the time range and the number of patients with missing corresponding appointments for both Advanced Bionics™ and Cochlear™.

Table S1a: Overview of appointments of patients with a Cochlear CI (N =89)

	T1 (switch on)	T2 (2 weeks)	T3 (1 month)	T4 (3 months)	T5 (6 months)	T6 (12 months)
n (within range)	89	78	70	76	65	71
n (outside of range)		11	15	5	2	9
n (no appointment)	0	0	4	8	22	9

Table S1b: Overview of appointments of patients with an Advanced Bionics CI (N = 94)

	T1 (switch on)	T2 (2 weeks)	T3 (1 month)	T4 (3 months)	T5 (6 months)	T6 (12 months)
n (within range)	94	88	84	86	75	86
n (outside of range)		6	8	6	0	3
n (no appointment)	0	0	2	2	19	5
