# Supplementary material for: Longitudinal Analysis of Cochlear Implant Stimulation Levels in the First Year: Optimizing Adult Fitting
Source: Ear Hear. 2026 Aug 19;47(6):1685–91. doi: 10.1097/AUD.0000000000001875 (PMC13712652; doi:10.1097/AUD.0000000000001875)
Supplement: Supplementary file 2 [file aud-47-1685-s002.pdf]

Table S2a and S2b show the results of the post-hoc tests for mean M- and T-levels of AB and Cochlear™ implant users. Significant values ($p < .05$) are highlighted in bold.

Table S2a: Linear mixed model pairwise comparisons for Advanced Bionics™.

	M-level			T-level		
Contrast	Estimate	Standard error	p-value	Estimate	Standard error	p-value
T1 - T2	-34.7	1.96	**< .001**	-14.4	1.41	**< .001**
T1 - T3	-58.5	2.32	**< .001**	-19.0	1.70	**< .001**
T1 - T4	-68.1	2.78	**< .001**	-21.0	2.06	**< .001**
T1 - T5	-71.2	3.39	**< .001**	-22.6	2.54	**< .001**
T1 - T6	-71.3	3.93	**< .001**	-22.5	2.96	**< .001**
T2 - T3	-23.8	1.98	**< .001**	-4.6	1.42	**0.015**
T2 - T4	-33.4	2.31	**< .001**	-6.6	1.69	**0.001**
T2 - T5	-36.5	2.86	**< .001**	-8.2	2.12	**0.002**
T2 - T6	-36.6	3.34	**< .001**	-8.1	2.51	**0.020**
T3 - T4	-9.6	1.99	**< .001**	-2.0	1.43	0.728
T3 - T5	-12.7	2.43	**< .001**	-3.5	1.77	0.341
T3 - T6	-12.8	2.82	**< .001**	-3.4	2.09	0.567
T4 - T5	-3.1	2.12	0.693	-1.5	1.51	0.910
T4 - T6	-3.2	2.35	0.757	-1.4	1.72	0.959
T5 - T6	-0.1	2.16	1.000	0.1	1.55	1.000

Table S2b: Linear mixed model pairwise comparisons for Cochlear™.

	C-level			T-level		
Contrast	Estimate	Standard error	p-value	Estimate	Standard error	p-value
T1 - T2	-19.3	0.94	**< .001**	-9.3	0.98	**< .001**
T1 - T3	-29.2	1.09	**< .001**	-13.8	1.07	**< .001**
T1 - T4	-33.5	1.32	**< .001**	-15.4	1.22	**< .001**
T1 - T5	-34.6	1.63	**< .001**	-16.2	1.45	**< .001**
T1 - T6	-34.7	1.86	**< .001**	-15.9	1.58	**< .001**
T2 - T3	-10.0	0.94	**< .001**	-4.6	0.99	**< .001**
T2 - T4	-14.2	1.11	**< .001**	-6.1	1.10	**< .001**
T2 - T5	-15.3	1.39	**< .001**	-6.9	1.30	**< .001**
T2 - T6	-15.4	1.60	**< .001**	-6.6	1.41	**< .001**
T3 - T4	-4.3	0.97	**< .001**	-1.5	1.02	0.670
T3 - T5	-5.4	1.19	**< .001**	-2.4	1.18	0.347
T3 - T6	-5.5	1.35	**0.001**	-2.0	1.26	0.588
T4 - T5	-1.1	1.07	0.915	-0.8	1.13	0.976
T4 - T6	-1.2	1.15	0.912	-0.5	1.15	0.998
T5 - T6	-0.1	1.08	1.000	0.3	1.15	1.000
