# Supplementary material for: Longitudinal Analysis of Cochlear Implant Stimulation Levels in the First Year: Optimizing Adult Fitting
Source: Ear Hear. 2026 Aug 19;47(6):1685–91. doi: 10.1097/AUD.0000000000001875 (PMC13712652; doi:10.1097/AUD.0000000000001875)
Supplement: Supplementary file 3 [file aud-47-1685-s003.pdf]

Table S3a and S3b show the results of the post-hoc tests for mean M- and T-levels per segment (apical, medial, upper basal, lower basal) of patients with an Advanced Bionics™ CI. Significant values ($p < .05$) are highlighted in bold.

Table S3a: Linear mixed model pairwise comparisons of M-levels per segment of patients with an Advanced Bionics™ CI.

	Apical			Medial			Upper-basal			Lower-basal		
Appointment	Estimate	p-value	Standard error	Estimate	p-value	Standard error	Estimate	p-value	Standards error	Estimate	p-value	Standard error
T1 - T2	-34.7	**< .001**	1.89	-36.6	**< .001**	2.50	-34.7	**< .001**	2.24	-32.2	**< .001**	2.45
T1 - T3	-58.9	**< .001**	2.21	-62.1	**< .001**	2.79	-58.7	**< .001**	2.74	-51.7	**< .001**	3.13
T1 - T4	-70.1	**< .001**	2.62	-73.0	**< .001**	3.16	-65.9	**< .001**	3.37	-58.4	**< .001**	3.99
T1 - T5	-74.4	**< .001**	3.18	-74.8	**< .001**	3.74	-69.0	**< .001**	4.17	-60.1	**< .001**	5.03
T1 - T6	-76.6	**< .001**	3.67	-76.6	**< .001**	4.19	-67.3	**< .001**	4.90	-57.7	**< .001**	6.01
T2 - T3	-24.3	**< .001**	1.91	-25.4	**< .001**	2.52	-23.9	**< .001**	2.27	-19.6	**< .001**	2.50
T2 - T4	-35.4	**< .001**	2.20	-36.4	**< .001**	2.77	-31.1	**< .001**	2.73	-26.2	**< .001**	3.15
T2 - T5	-39.7	**< .001**	2.70	-38.1	**< .001**	3.28	-34.2	**< .001**	3.46	-27.9	**< .001**	4.12
T2 - T6	-41.9	**< .001**	3.14	-39.9	**< .001**	3.66	-32.6	**< .001**	4.13	-25.6	**< .001**	5.04
T3 - T4	-11.1	**< .001**	1.92	-10.9	**< .001**	2.54	-7.2	**0.020**	2.27	-6.7	0.086	2.51
T3 - T5	-15.5	**< .001**	2.31	-12.7	**< .001**	2.94	-10.3	**0.005**	2.85	-8.4	0.117	3.30
T3 - T6	-17.6	**< .001**	2.66	-14.5	**< .001**	3.22	-8.7	0.117	3.42	-6.0	0.685	4.10
T4 - T5	-4.4	0.265	2.03	-1.8	0.986	2.69	-3.1	0.787	2.40	-1.7	0.989	2.67
T4 - T6	-6.5	0.045	2.24	-3.5	0.812	2.83	-1.5	0.995	2.78	0.6	1.000	3.26
T5 - T6	-2.1	0.908	2.08	-1.8	0.988	2.75	1.6	0.986	2.47	2.3	0.961	2.79

Table S3b: Linear mixed model pairwise comparisons of T-levels per segment of patients with an Advanced Bionics CI.

	Apical			Medial			Upper-basal			Lower-basal		
Appointment	Estimate	Standard error	p-value	Estimate	Standard error	p-value	Estimate	Standard error	p-value	Estimate	Standard error	p-value
T1 - T2	-14.0	1.08	**< .001**	-13.253	1.04	**< .001**	-13.307	1.91	**< .001**	-20.053	1.26	**< .001**
T1 - T3	-18.853	1.16	**< .001**	-17.733	1.15	**< .001**	-17.956	1.94	**< .001**	-24.596	1.44	**< .001**
T1 - T4	-22.048	1.31	**< .001**	-19.777	1.34	**< .001**	-19.159	2.04	**< .001**	-24.729	1.71	**< .001**
T1 - T5	-23.843	1.55	**< .001**	-21.472	1.61	**< .001**	-20.032	2.26	**< .001**	-26.902	**2.10**	**< .001**
T1 - T6	-24.664	1.68	**< .001**	-22.042	1.79	**< .001**	-20.109	2.22	**< .001**	-27.124	**2.37**	**< .001**
T2 - T3	-4.844	1.08	**0.003**	-4.480	1.04	**0.025**	-4.649	1.91	0.058	-4.543	1.27	0.385
T2 - T4	-8.039	1.19	**< .001**	-6.524	1.18	**0.002**	-5.852	1.99	**0.046**	-4.676	1.47	0.592
T2 - T5	-9.834	1.40	**< .001**	-8.218	1.42	**0.002**	-6.725	2.20	0.100	-6.849	1.82	0.449
T2 - T6	-10.655	1.50	**< .001**	-8.788	1.57	**0.008**	-6.802	2.14	0.248	-7.071	2.05	0.629
T3 - T4	-3.195	1.11	0.145	-2.044	1.07	0.722	-1.202	1.97	0.979	-0.133	1.31	1.000
T3 - T5	-4.990	1.29	0.020	-3.739	1.27	0.292	-2.075	2.16	0.922	-2.306	1.59	0.974
T3 - T6	-5.811	1.35	0.017	-4.309	1.38	0.314	-2.153	2.09	0.959	-2.528	1.77	0.984
T4 - T5	-1.795	1.24	0.788	-1.694	1.20	0.880	-0.873	2.19	0.996	-2.173	1.47	0.956
T4 - T6	-2.616	1.25	0.519	-2.265	1.24	0.781	-0.951	2.08	0.997	-2.395	1.56	0.968
T5 - T6	-0.821	1.26	0.992	-0.570	1.22	0.999	-0.078	2.21	1.000	-0.222	1.50	1.000
