# Supplementary material for: Longitudinal Analysis of Cochlear Implant Stimulation Levels in the First Year: Optimizing Adult Fitting
Source: Ear Hear. 2026 Aug 19;47(6):1685–91. doi: 10.1097/AUD.0000000000001875 (PMC13712652; doi:10.1097/AUD.0000000000001875)
Supplement: Supplementary file 4 [file aud-47-1685-s004.pdf]

Table S4a and S4b show the results of the post-hoc tests for C- and T-levels per segment (apical, medial, upper basal, lower basal) of patients with a Cochlear™ CI. Significant values ($p < .05$) are highlighted in bold.

Table S4a: Linear mixed model pairwise comparisons of C-levels per segment of patients with a Cochlear™ CI.

	Apical			Medial			Upper-basal			Lower-basal		
Appointment	Estimate	Standard error	p-value	Estimate	Standard error	p-value	Estimate	Standard error	p-value	Estimate	Standard error	p-value
T1 - T2	-20.6	1.01	**< .001**	-21.8	1.02	**< .001**	-19.3	1.05	**< .001**	-12.4	1.18	**< .001**
T1 - T3	-31.6	1.15	**< .001**	-32.4	1.20	**< .001**	-28.7	1.21	**< .001**	-19.2	1.41	**< .001**
T1 - T4	-37.4	1.36	**< .001**	-36.7	1.47	**< .001**	-32.5	1.45	**< .001**	-20.1	1.73	**< .001**
T1 - T5	-39.9	1.66	**< .001**	-37.8	1.82	**< .001**	-32.6	1.78	**< .001**	-19.0	2.15	**< .001**
T1 - T6	-40.7	1.89	**< .001**	-37.7	2.09	**< .001**	-32.2	2.02	**< .001**	-18.8	2.47	**< .001**
T2 - T3	-11.0	1.01	**< .001**	-10.6	1.02	**< .001**	-9.4	1.05	**< .001**	-6.8	1.19	**< .001**
T2 - T4	-16.9	1.17	**< .001**	-14.9	1.22	**< .001**	-13.2	1.23	**< .001**	-7.7	1.44	**< .001**
T2 - T5	-19.3	1.44	**< .001**	-16.0	1.54	**< .001**	-13.3	1.53	**< .001**	-6.5	1.82	**0.005**
T2 - T6	-20.1	1.62	**< .001**	-15.9	1.78	**< .001**	-12.9	1.74	**< .001**	-6.4	2.12	**0.034**
T3 - T4	-5.8	1.04	**< .001**	-4.3	1.05	**0.001**	-3.7	1.08	**0.008**	-0.9	1.23	0.980
T3 - T5	-8.3	1.26	**< .001**	-5.4	1.31	**0.001**	-3.8	1.32	**0.044**	0.3	1.54	1.000
T3 - T6	-9.08	1.39	**< .001**	-5.331	1.50	**0.006**	-3.414	1.48	0.197	0.400	1.77	1.000
T4 - T5	-2.457	1.16	0.279	-1.069	1.17	0.943	-0.098	1.21	1.000	1.127	1.38	0.964
T4 - T6	-3.251	1.22	0.083	-0.994	1.27	0.970	0.332	1.28	1.000	1.276	1.50	0.957
T5 - T6	-0.794	1.17	0.984	0.075	1.18	1.000	0.430	1.21	0.999	0.149	1.39	1.000

Table S4b: Linear mixed model pairwise comparisons of T-levels per segment of patients with a Cochlear™.

	Apical			Medial			Upper-basal			Lower-basal		
Appointment	Estimate	Standard error	p-value	Estimate	Standard error	p-value	Estimate	Standard error	p-value	Estimate	Standard error	p-value
T1 - T2	-10.3	1.08	**< .001**	-10.6	1.04	**< .001**	-8.8	1.91	**< .001**	-6.3	1.26	**0.001**
T1 - T3	-15.1	1.16	**< .001**	-16.3	1.15	**< .001**	-13.5	1.92	**< .001**	-8.3	1.44	**< .001**
T1 - T4	-18.2	1.31	**< .001**	-17.9	1.34	**< .001**	-14.5	2.04	**< .001**	-6.6	1.71	**0.002**
T1 - T5	-19.1	1.55	**< .001**	-18.3	1.61	**< .001**	-18.3	2.26	**< .001**	-5.3	2.09	0.120
T1 - T6	-20.4	1.68	**< .001**	-19.0	1.79	**< .001**	-14.0	2.22	**< .001**	-4.4	2.37	0.439
T2 - T3	-4.8	1.08	**0.002**	-5.7	1.04	**< .001**	-4.7	1.91	0.138	-2.0	1.27	0.616
T2 - T4	-7.9	1.19	**< .001**	-7.3	1.18	**< .001**	-5.7	1.99	0.050	-0.3	1.47	1.000
T2 - T5	-8.8	1.40	**< .001**	-7.7	1.42	**< .001**	-9.6	2.20	**0.000**	1.0	1.82	0.994
T2 - T6	-10.1	1.50	**< .001**	-8.4	1.57	**< .001**	-5.2	2.14	0.145	2.0	2.05	0.934
T3 - T4	-3.1	1.11	0.058	-1.6	1.07	0.691	-1.0	1.97	0.996	1.7	1.31	0.776
T3 - T5	-4.0	1.29	**0.024**	-2.0	1.27	0.618	-4.9	2.16	0.219	3.0	1.59	0.409
T3 - T6	-5.3	1.35	**0.002**	-2.7	1.38	0.388	-0.5	2.09	1.000	4.0	1.77	0.231
T4 - T5	-0.9	1.24	0.979	-0.4	1.20	0.999	-3.9	2.19	0.485	1.3	1.47	0.952
T4 - T6	-2.18	1.25	0.503	-1.1	1.24	0.950	0.5	2.08	1.000	2.2	1.56	0.711
T5 - T6	-1.29	1.26	0.910	-0.7	1.22	0.994	4.3	2.21	0.370	0.9	1.50	0.990
